# Stem cell-derived exosomes repair ischemic muscle injury by inhibiting the tumor suppressor Rb1-mediated NLRP3 inflammasome pathway

**DOI:** 10.1038/s41392-021-00520-8

**Published:** 2021-03-17

**Authors:** Yanli Wang, Wenping Xie, Bin Liu, Hui Huang, Wei Luo, Yu Zhang, Xiangbin Pan, Xi-Yong Yu, Zhenya Shen, Yangxin Li

**Affiliations:** 1 Institute for Cardiovascular Science and Department of Cardiovascular Surgery, First Affiliated Hospital and Medical College of Soochow University, Collaborative Innovation Center of Hematology, Soochow University, Suzhou, Jiangsu PR China; 2 Department of Cardiology, The Second Hospital of Jilin University, Changchun, Jilin PR China; 3 Cardiovascular Department, The Eighth Affiliated Hospital, Sun Yat-sen University, Shenzhen, Guangdong PR China; 4 Department of Cardiac Surgery, Fuwai Hospital, Beijing, PR China; 5 Key Laboratory of Molecular Target & Clinical Pharmacology and the State Key Laboratory of Respiratory Disease, Guangzhou Medical University, Guangzhou, Guangdong PR China

**Keywords:** Mesenchymal stem cells, Translational research, Non-coding RNAs

**Dear Editor,**

Lower-limb ischemia is a serious clinical condition affecting many patients world-wide and there is no effective therapy. Ischemia activates the NLRP3 inflammasome, which triggers tissue damage by releasing inflammatory cytokines including IL-1β and IL-18.^1^

However, the molecular mechanisms underlying activation of the NLRP3 inflammasome remain largely unknown.

We used RNA sequencing (RNAseq) to compare the mRNA expression in ischemic and normal muscles. RNAseq revealed increased mRNA levels of retinoblastoma-1 (Rb1), NLRP3 inflammasome, IL-1β, and IL-18 (Fig. 1a, b), and this was confirmed by qRT-PCR (Fig. 1c). These findings were also verified at the protein level by Western blot (Fig. 1d) and ELISA (Fig. 1e). Gene ontology analysis of the upregulated genes revealed biological process terms associated with the inflammatory response pathway and the NLRP3 inflammasome in ischemic muscle, confirming that inflammation-related signaling pathways are involved in ischemia-induced muscle injury (Fig. 1f). To verify the data from RNAseq, we also performed protein sequencing. Heat map and Spearman’s correlation analysis revealed a good protein–mRNA correlation of Rb1, NLRP3, IL-1β, and IL-18 in ischemic muscle (Supplementary Fig. 1a–e).

**Fig. 1:**
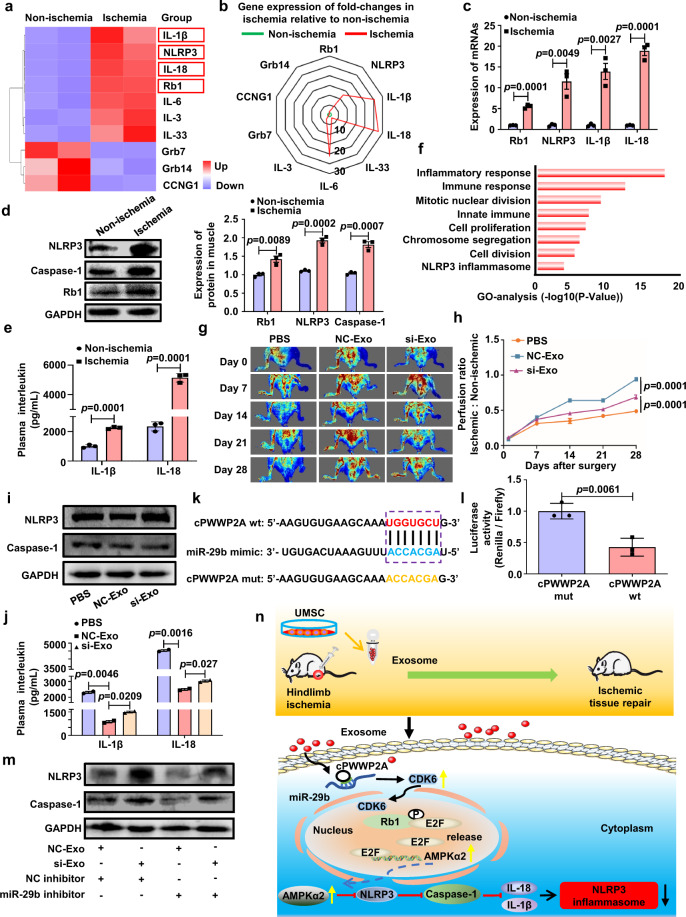
Tumor-suppressor Rb1 is an NLRP3 inflammasome-inducer in skeletal muscle. **a** Heatmap of mRNA sequencing data from ischemic and control groups (blue, downregulated; red, upregulated). **b** Radar chart of mRNA sequencing data from ischemic and control groups (higher number indicates upregulation). **c** qRT-PCR analysis of mRNA levels of Rb1, NLRP3, IL-1β, and IL-18 in non-ischemic and ischemic muscles. **d** Western blot analysis showing increased expressions of NLRP3, Caspase1, and Rb1 protein in muscles after ischemic injury. **e** The levels of IL-1β and IL-18 in plasma from control and ischemia mice were assessed by ELISA. **f** Top 8 significant enrichment of GO term of differentially regulated genes in ischemic muscle. **g** Laser Doppler perfusion imaging of limbs from mice treated with PBS, NC-Exo, or si-Exo at different time points after ischemic surgery. **h** Blood flow recovery after treatment with PBS, NC-Exo, or si-Exo. **i** Western blot analysis of NLRP3 and Caspase-1 proteins in the muscles treated with PBS, NC-Exo, or si-cPWWP2A (si-Exo). **j** ELISA analysis of plasma IL-1β and IL-18 levels from mice treated with PBS, NC-Exo, or si-Exo. **k** Illustration of putative complementary sites within cPWWP2A and miR-29b by CircBank analysis. **l** Dual-luciferase reporter assay demonstrating interaction between cPWWP2A and miR-29b. **m** Western blot analysis showing that inhibition of miR-29b in C2C12 cells results in the reduced expression of NLRP3 and Caspase-1, and the effects are reversed by cPWWP2A silencing in exosomes. **n** Schematic illustration of the circPWWP2A/Rb1/AMPKα2/NLRP3 pathway mediating the beneficial effect of exosomes. Data are presented as the mean ± SD

Rb1 protein is a known tumor-suppressor, and inactivation of Rb1 promotes the proliferation of skeletal muscle cells,^2^ but the role of Rb1 in ischemic muscle injury remains unknown. To address this question, we generated muscle-specific Rb1-knockout (Rb1-mKO) mice (Supplementary Fig. 2a–e). After ischemic hindlimb injury, Rb1-mKO mice showed faster recovery of blood flow than control mice (Supplementary Fig. 3a, b). The hindlimb grip strength, endurance, and the ratio of skeletal muscle to body weight were also higher in Rb1-mKO mice at 28 days after injury (Supplementary Fig. 3c–e). These results indicate that loss of the Rb1 gene promotes the repair of skeletal muscle after injury. Moreover, the protein expression of NLRP3 and Caspase-1 in muscle, as well as the plasma levels of IL-1β and IL-18 were lower in Rb1-mKO mice than in control mice (Supplementary Fig. 4a, b), suggesting that deletion of the Rb1 gene results in inhibition of the NLRP3 signaling pathway. To confirm these findings, we established an in vitro model of inflammasome activation by stimulating C2C12 myoblasts with lipopolysaccharide (LPS) and ATP. Knockdown of Rb1 (Rb1-siRNA) enhanced cell proliferation (Supplementary Fig. 4c), decreased the activation of NLRP3 and Caspase-1 (Supplementary Fig. 4d), and reduced the levels of IL-1β and IL-18 (Supplementary Fig. 4e).

We used the STRING database to identify the upstream signaling molecules that regulate Rb1, and found that it interacts with CDK6 and the transcription factor E2F (Supplementary Fig. 5a). Previous studies have shown that miR-29b targets CDK6,^3^ so we investigated whether miR-29b regulates CDK6/Rb1. The 3’UTR of CDK6 mRNA and miR-29b has multiple binding sites as shown by Targetscan, and dual luciferase experiments confirmed that CDK6 is a target of miRNA-29b (Supplementary Fig. 5b). Moreover, an miR-29b mimic inhibited the expression of CDK6 protein and the phosphorylation of Rb1 protein in C2C12 cells (Supplementary Fig. 5c).

To determine how miR-29b was regulated, we used circRNA sequencing to compare circRNA expression in ischemic and normal muscle. Several circRNAs (cPWWP2A, circ_CCDC66, and circ_HECTD1) that promote cell proliferation were detected in ischemic muscle (Supplementary Fig. 6a). Among these circRNAs, cPWWP2A had the strongest expression, which was dramatically decreased under ischemic conditions. These findings were confirmed by qRT-PCR (Supplementary Fig. 6b, c).

Since stem cell-derived exosomes can promote tissue repair, we injected either control exosomes (NC-Exos) or exosomes with cPWWP2A silencing (si-Exos) into ischemic muscles to determine whether cPWWP2A was involved in mediating the beneficial effect of exosomes from human umbilical cord-derived mesenchymal stem cells (UMSC-Exos) in muscle. NC-Exos treatment led to increased expression of cPWWP2A in muscles, which was attenuated by si-Exos (Supplementary Fig. 7a). Compared to mice injected with NC-Exos, si-Exos treatment led to slower recovery of blood flow (Fig. 1g, h), poor motor function, reduced muscle force and running distance (Supplementary Fig. 7b–e), a reduced muscle to body weight ratio (Supplementary Fig. 7f), higher expression of NLRP3, Caspase-1 (Fig. 1i), and increased plasma levels of IL-1β and IL-18 (Fig. 1j). The injected exosomes have a diameter of 30 nm–100 nm (Supplementary Fig. 7g), and were only detected in the muscle (Supplementary Fig. 7h). The expression of NLRP3 and Caspase-1 was significantly higher in C2C12 cells which were transfected with si-Exos compared with cells transfected with NC-Exos (Supplementary Fig. 8a). Consequently, the levels of IL-1β and IL-18 were also increased (Supplementary Fig. 8b, c), and cell proliferation was inhibited by si-Exos treatment (Supplementary Fig. 8d).

We identified an interaction between miR-29b and cPWWP2A based on CircBank analysis (Fig. 1k). The link between cPWWP2A and miR-29b was confirmed by dual-luciferase assays showing a strong binding of miR-29b to cPWWP2A (Fig. 1l). The expression of miR-29b was increased in ischemic muscle (Supplementary Fig. 9a), which was contrary to that of cPWWP2A. These results suggest that cPWWP2A is a molecular sponge for miR-29b. To verify that cPWWP2A/miR-29b regulates the NLRP3 signaling pathway, C2C12 cells were treated with an miR-29b inhibitor, which reduced the expression of NLRP3 and Caspase-1 as well as the release of IL-1β and IL-18, but enhanced C2C12 cell proliferation, and these effects were reversed by si-Exos (Fig. 1m and Supplementary Fig. 9b–d). Accordingly, knockdown of cPWWP2A in C2C12 cells resulted in a decrease in the expression of CDK6 mRNA (Supplementary Fig. 9e) and protein (Supplementary Fig. 9f). Moreover, the differentiation of C2C12 cells was inhibited by cPWWP2A siRNA (Supplementary Fig. 9g), but was stimulated by miR-29b inhibitor (Supplementary Fig. 9h). These data indicate that cPWWP2A regulates Rb1 *via* the miR-29b/CDK6 pathway.

Previous studies have shown that downregulation of Rb1 leads to the release of E2F transcription factor, which in turn activates the transcription of AMP kinase α2 (AMPKα2).^4^

AMPKα2 inhibits the activation of NLRP3 in smooth muscle cells.^5^ We sought to determine whether Rb1 regulates the expression of AMPKα2 in skeletal muscle. Our data showed that the expression of AMPKα2 mRNA and protein was increased in Rb1-mKO mice (Supplementary Fig. 10a, b). AMPKα2 silencing resulted in increased expression of NLRP3 and Caspase-1 (Supplementary Fig. 10c–e), increased release of IL-1β and IL-18 into the cell supernatant (Supplementary Fig. 10f), and decreased cell proliferation (Supplementary Fig. 10g). Moreover, the injection of AMPKα2 siRNA in muscle increased the mRNA expression of NLRP3, Caspase-1, IL -1β and IL-18 in muscle (Supplementary Fig. 10h–i), the protein expression of NLRP3 and Caspase-1 (Supplementary Fig. 10j), and the plasma levels of IL -1β and IL-18 in mice (Supplementary Fig. 10k). These data indicate that downregulation of Rb1 inhibits the activation of NLRP3 by activating AMPKα2.

In summary, Rb1 protein is a known tumor suppressor, but we show that it acts as an NLRP3 inflammasome-inducer in skeletal muscle. We further demonstrate that downregulation of Rb1 blocks the activation of NLRP3 inflammation *via* the E2F/AMPKα2-mediated signaling pathway. Rb1 is inactivated by CDK6, which is a downstream target of miR29b. In ischemic muscle, the levels of miR29b are increased, leading to reduced expression of CDK6 and over-activation of Rb1. cPWWP2A represses the activity of miR29b by binding with it. Therefore, we have deciphered a new mechanism that regulates the NLRP3 inflammasome through the cPWWP2A/Rb1/AMPKα2/NLRP3 signaling pathway (Fig. 1n). Importantly, we show that UMSC-Exos can supplement the loss of cPWWP2A in ischemic muscle. Thus, the Rb1-knockout approach can be translated into clinical application by using UMSC-Exos, which promote muscle repair by releasing cPWWP2A.

## Supplementary information

Stem cell-derived exosomes repair ischemic muscle injury by inhibiting the tumor suppressor Rb1-mediated NLRP3 inflammasome pathway

## Data Availability

All supporting data are included in the manuscript and Supplemental files. Additional data are available upon reasonable request to the corresponding author.
