# Supplementary material for: Stem cell-derived exosomes repair ischemic muscle injury by inhibiting the tumor suppressor Rb1-mediated NLRP3 inflammasome pathway
Source: Signal Transduct Target Ther. 2021 Mar 17;6:121. doi: 10.1038/s41392-021-00520-8 (PMC7966359; doi:10.1038/s41392-021-00520-8)
Supplement: Supplementary file 1 — Stem cell-derived exosomes repair ischemic muscle injury by inhibiting the tumor suppressor Rb1-mediated NLRP3 inflammasome pathway [file 41392_2021_520_MOESM1_ESM.doc]

**Supplementary Materials for**

**Stem cell-derived exosomes repair ischemic muscle injury by inhibiting the tumor suppressor Rb1-mediated NLRP3 inflammasome pathway**

Yanli Wang^1#^, Wenping Xie^1#^, Bin Liu^2#^, Hui Huang^3#^, Wei Luo^1^, Yu Zhang^1^, Xiangbin Pan^4^, Xi-Yong Yu^5^, Zhenya Shen^1*^, Yangxin Li^1*^

Correspondence to: yangxin_li@yahoo.com (YL) or [uuzyshen@aliyun.com](mailto:uuzyshen@aliyun.com) (ZS)

**This PDF file includes:**

Materials and Methods

Figures. S1 to S10

**Materials and Methods**

**Harvesting and identification of UMSC exosomes**

Exosomes were harvested and identified as we reported previously.^1^ UMSCs (purchased from Jiangsu Heze Biotechnology Co., Ltd, China) were cultured in α-minimum essential medium containing 10% fetal bovine serum (FBS), which had been centrifuged at 100,000 g to eliminate preexisting bovine-derived exosomes. After 48 h in culture, exosomes were isolated from the culture supernatant using a total exosome isolation kit (Life Technology, San Francisco, CA, USA). The culture medium collected from UMSCs was centrifuged at 2,000 g for 30 min to remove dead cells and debris, and then transferred to a new tube containing 0.5 volumes of the Total Exosome Isolation reagent. The mixture was incubated at 4℃ overnight and centrifuged at 10,000 g for 1 h at 4℃. The pellet was re-suspended in PBS and stored at −80℃. The protein concentration of exosomes was determined using a BCA protein assay kit (Takara, Japan). Because exosomes are not large enough to be detected directly by flow cytometry, they were pre-bound to aldehyde/sulfate latex beads (4 μm; Molecular Probes; Invitrogen) to amplify the channel signal as we have described previously. ^1^ The exosomes were then incubated with a fluorescein isothiocyanate conjugated antibody against the exosome surface marker CD63, and analyzed by flow cytometry. The expression of CD63 was also analyzed by Western blot.

**Nanoparticle size analysis**

Exosome size and number of particles were assessed using a NanoSight NS300 equipped with a 405 nm laser (Malvern, Great Malvern, UK). Videos (60 s duration, 30 frames/sec) were recorded and particle movement was analyzed using the NTA software (NanoSight version 2.3).

**Detection of PKH26-labeled exosomes**

PKH26-labeled exosomes were re-suspended in PBS. Exosomes were injected into the muscle of each mouse at three injection points. The muscles were isolated and washed twice with PBS, fixed, and nuclei were stained with 4′,6-diamidino-2-phenylindole (DAPI). The frozen muscle sections were analyzed using an inverted fluorescence microscope (Olympus, Tokyo, Japan).

**Myotube staining**

C2C12 cells were cultured in Dulbecco’s modified Eagle’s medium (DMEM) containing with 10% fetal bovine serum at 37℃ with 5% CO_2_. To induce differentiation, the medium was switched into differentiation medium (DMEM containing 2% horse serum) and cultured for 4 days. Myotube transfection was performed with Lipofectamine2000 Reagent (Invitrogen) according to the manufacturer’s instructions. The inhibitor negative control and miR-29b inhibitor were purchased from RiboBio. The transfection dosage of miR-29b inhibitor, AMPKα2 siRNA, cPWWP2A siRNA, and their respective controls were 100 nM. After the transfection, the cells were cultured in differentiation medium for 4 days. C2C12 myotubes were fixed by 4% PFA for 30 min at room temperature, permeabilized with 0.5% Triton X-100 in PBS for 15 min, and then blocked with 5% BSA in PBST for 1 h at room temperature. Myotubes were incubated with anti-MHC (MF-20, 1:100, DSHB) diluted in 5% BSA overnight at 4℃. After washing, myotubes were incubated with secondary antibody Goat Anti-Mouse IgG H&L (1:500, ab150113, Alexa Fluor® 488, Abcam) for 1 h at room temperature. Nuclear staining was performed with DAPI.

**Generation of muscle-specific Rb1-knockout (Rb1-mKO) mice**

To generate conditional Rb1-mKO mice (Rb1^flox/Wt^), a construct was engineered for conditional disruption of the Rb1 gene, with two loxP sites flanking the third exon of the Rb1 gene. We constructed a vector targeting ES cells that contained a 4.0-kb 5 'homology arm, a 0.7-kb flox region, PGK-Neo-polyA, a 4.0-kb 3' homology arm, and an MC1-TK-polyA negative selection marker. After the vector was linearized, ES cells were transfected electrically. Positive clones were selected by G418 screening and verified by long-fragment PCR identification. Positive clones with correct homologous recombination were expanded and injected into blastocysts of C57BL/6J mice to obtain chimeric mice (Rb1^flox/Wt^). The Rb1^flox/Wt^ mice were generated by the Shanghai Model Organisms Center, Inc.

Muscle-specific Rb1-mKO mice were generated by crossing Pax7 ^Cre^**/**^wt^ mice with Rb1^flox/flox^ mice. Then, heterozygous Pax7 ^Cre^**/**wt/Rb1^flox/wt^ mice were back-crossed with the Rb1^flox/flox^ mice to obtain muscle-specific Rb1-mKO mice. All animal procedures were conducted in accordance with the Guidelines or the Care and Use of Laboratory Animals and were approved by the Institutional Animal Care and Use Committee at Soochow University (Suzhou, China).

**RNA extraction and real-time PCR**

RNA was extracted using TRIzol reagent (Takara). Total RNA was reverse-transcribed using a PrimeScript RT reagent kit (Takara). Real-time PCR was performed using a SYBR Premix Ex Taq kit (Takara) and the Applied Biosystems 7500 Real-Time PCR System (ABI, CA, USA) with the following primers:

mouse GAPDH: forward, 5'-AAATGGTGAAGGTCGGTGTG-3', reverse, 5'-TGAAGGGGTCGTTGATGG-3';

human cPWWP2A: forward, 5'-CAGTCTCGCTGCACCTCTAC-3', reverse, 5'-GCGGCATGGCTTCTGGTTTA-3';

mouse cPWWP2A: forward, 5'-ACTGGAGAAAATTCGGAGT-3', reverse, 5'-TCCGGTTTGTCCTTATATTC-3';

human PWWP2A mRNA: forward, 5'-TGCCGCTCCAAAGTAATACA-3', reverse, 5'-CATGGAAGAGAGGTGGTGGT-3';

human GAPDH: forward, 5'-CAACGGGAAACCCATCACCAT-3', reverse, 5'-AGATGATGACCCTTTTGGCCCC-3'.

To quantify circRNA and miRNA, GAPDH and U6 were respectively used as reference genes. The circRNA/mRNA reaction conditions were: 95℃, 30 s pre-denaturation, and 95℃ 5s, 60℃ 30 s repeated for 40 cycles. The reaction conditions for miRNA qPCR were: 95℃, 10 min pre-denaturation, and 95℃ 2 s, 60℃ 30 s, repeated for 40 cycles. Three replicate wells were set for each gene, the expression of genes was calculated by the 2^–ΔΔCt^ method, and each experiment was repeated three times.

**Western blot**

Cells were washed with PBS and lysed in lysis buffer on ice for 30 min. After centrifugation at 12,000 g for 10 min, the protein content of the supernatant was determined using a BCA kit. The protein extracts were separated by polyacrylamide gel electrophoresis (12%) and transferred to polyvinylidene difluoride membranes. The primary antibodies against NLRP3 (catalog # ab214185), Caspase-1 (catalog # ab1872), IL-1β (catalog # ab9722), and p-Rb1(catalog # ab47763) were from Abcam (Cambridge, UK). Antibodies against GAPDH (catalog # Mab5465-100) and HRP-linked anti-rabbit Immunoglobulin G (IgG) (catalog # GAR007) were from MultiSciences Biotech Co. (Hangzhou, China). The primary antibody against CD63 (catalog # ab134045) was from Abcam (Cambridge, MA, USA). The antibodies against CDK6 (catalog # 3136s) and AMPKα2 (catalog # 5831s) were from Cell Signaling Technology (Boston, MA, USA). Another primary antibody against CD63 (catalog # sc-5275) was from Santa Cruz Biotechnology (Dallas, TX, USA). The antibodies were diluted in NCM Universal Antibody Diluent (catalog # WB100D), protein signals were detected using an ECL chemiluminescence kit (catalog # P10200) from New Cell & Molecular Biotech Co., Ltd (Suzhou, China), and the luminescence was visualized using a BioRad luminescent imaging system.

**Mouse unilateral hindlimb ischemia model**

Adult male 8–12-week-old C57BL/6 mice were supplied by the Experimental Animal Center of Soochow University (Suzhou, China). The animal experiments were approved by the Animal Care and Use Committee of Soochow University. The mice were randomly assigned to three treatment groups, PBS, exosomes, and si-circPWWP2A exosomes. Unilateral mouse hindlimb ischemia was created by ligating the left femoral artery under general anesthesia (2–4% isoflurane in oxygen). Immediately after surgery, the muscle of the ischemic hindlimb was injected with one of the above treatments; 100 μg exosomes was injected into the muscle of each mouse at three points.

**Laser Doppler perfusion imaging of mouse hindlimb**

A laser Doppler perfusion imager (LDPI, Moor Instruments, Axminster, UK) was used to monitor blood flow in the hindlimb at 1, 7, 14, 21, and 28 days after surgery. Perfusion was expressed as the ratio of the ischemic over the contralateral, non-manipulated leg.

**Running endurance**

On day 28 after surgery, each group of mice was exercised following a run-to-exhaustion protocol. Prior to running, each mouse was acclimated to the treadmill (Jiangsu SANS Biological Technology Co. Ltd) for 1–2 h and to the motor sound for 15 min. The belt was initially set at a slow speed (6 m/min), then the velocity was increased 2 m/min every 2 min for the first 12 min and then held steady (18 m/min). Exhaustion was defined as the point when mice spent >10 s on the shock grid without seeking to re-engage the treadmill.

**Muscle force measurement**

At day 28 after surgery, grip strength was measured using a Grip Strength Meter (Ji-Nan Biotechnology, Shandong, China). Five grip tests were run at 1-min intervals, and the average was calculated.

**Cell culture and the loss/gain-of-function approach**

Mouse myoblast C2C12 cells were cultured in Dulbecco’s modified Eagle’s medium (DMEM) supplemented with 10% FBS in 5% CO_2_ at 37℃. To induce differentiation, the medium was switched to DMEM containing 2% horse serum (Gibco, Cat # 1852632) when the density of cells reached 70–80%, and they were cultured for 6 days. To induce the formation of inflammasomes, C2C12 cells were incubated in medium containing *Escherichia coli* 0111:B4 LPS (200 ng/mL, Cat #: L8274, Sigma-Aldrich) for 24 h, followed by ATP (2.5 mM; Cat #:10519979001, Sigma-Aldrich) and incubated for 2 h.

The miR-29b mimic, miR-29b inhibitor, and small-interfering RNA (si-circPWWP2A) targeting circPWWP2A were synthesized by Ribobio (Guangzhou, China). The cells were transfected using Lipofectamine 2000 (Invitrogen, Carlsbad, CA, USA) according to the manufacturer’s instructions. For experiments involving Exo- si-circPWWP2A, the cells were transfected with si-circPWWP2A targeting circPWWP2A (CTACCCGCTCAGCAGGTTT) for 48 h, then the exosomes were isolated as described above.

An siRNA was designed to target the coding region of Rb1 mRNA (GTCAAGGGCTTACCATACT), another siRNA was designed to target the coding region of AMPKα2 (CCAATTGACAGGCCATAAA), and synthesized by Ribobio (Guangzhou, China). Scrambled siRNAs that did not lead to the degradation of any known cellular mRNA were used as negative controls. C2C12 cells (3 × 10^5^) were incubated with the siRNAs (100 nM) before experiments.

**Dual-luciferase reporter assay**

The firefly luciferase reporter psiCHECKTM-2 vector was from Promega, and the cPWWP2A sequence was cloned into a vector and named cPWWP2A-miR-29b-WT. A cPWWP2A sequence containing a miR-29b binding site mutation was synthesized by GENEWIZ Biological Technology Co., Ltd (Suzhou, China). It was cloned into a vector and named cPWWP2A-miR-29b-Mut. After the vector was constructed, it was verified by sequencing. The primer sequences used were: for the WT, forward: 5'-TCGACTTGTGACTAGGTTTACCACGAG-3', reverse: 5'-GGCCCTCGTGGTAAACCTAGTCACAAG-3'; and for the Mut, forward: 5'-TCGACAAGTGTGAAGCAAATGGTGCTG-3', reverse: 5'-GGCCCAGCACCATTTGCTTCACACTTG-3'. HEK293T cells were seeded in 96-well plates (5 ×10^3^ cells per well) and cultured for 24 h prior to transfection. The cells were co-transfected with a mixture of 50 ng reporter plasmid (cPWWP2A-miR-29b-WT or cPWWP2A-miR-29b-Mut), with 200 nM miR-29b mimic from RiboBio. After 48 h, the luciferase activity was analyzed using a dual luciferase reporter assay system (Promega, USA) per the manufacturer’s instructions.

**EdU proliferation assay**

Cell proliferation was assessed using an EdU Cell Proliferation Assay kit (RiboBio, Guangzhou, China). After different treatments, C2C12 cells were incubated in fresh medium containing 10 μM EdU for 2 h, then washed with PBS, fixed in 4% paraformaldehyde for 30 min, and treated with 0.5% Triton X-100 for 10 min. The nuclei were stained with Hoechst 3342 for 15 min. Finally, the proportion of cells incorporating EdU was determined by ﬂuorescence microscopy.

**Enzyme-linked immunosorbent assay (ELISA)**

The concentrations of serum and medium IL-1β and IL-18 were measured using an ELISA kit according to the manufacturer’s instructions. The IL-1β (Cat # EK201B/3) and IL-18 (Cat # EK218) ELISA kits were from MultiSciences (Lianke) Biotech Co., Ltd (Hangzhou, China).

**circRNA/mRNA sequencing and analysis**

Male 8-week-old C57BL/6 mice were randomly assigned to control (n = 3) and ischemic groups (n = 3). Unilateral hindlimb ischemia was created by ligating the left femoral artery under general anesthesia (2–4% isoflurane in oxygen). Total RNA from muscles was extracted by TRIzol reagent (Invitrogen), and cDNA was synthesized from ribosome-depleted RNA samples using random hexamer primers. Whole transcriptome sequencing data obtained from the HiseqTM Sequencer were filtered to remove the adaptor sequences, as were reads with >5% ambiguous bases, and low-quality reads containing >20% of bases with quality <20. The data were mapped to the mouse genome using HISAT2. HTSeq was used to calculate the gene counts of mRNA and circRNA. All RNA-seq and bioinformatics analyses were performed at NovelBio Ltd (Shanghai, China).

**Sample preparation for proteomics sequencing**

The tissues were ground in liquid nitrogen, and lysed in lysis buffer containing 7 M urea, 4% SDS, and protease inhibitor cocktail (Roche Ltd. Basel, Switzerland), followed by sonication on ice. The samples were centrifuged at 13,000 rpm for 10 min at 4℃ to remove insoluble particles. The supernatant was collected and the protein concentration was determined using the BCA protein assay. Aliquots of the supernatant containing 100 μg protein were transferred into a new tubes and the final volume was adjusted to 100 μL with 100 mM TEAB (triethylammonium bicarbonate). The samples were incubated with 5 μL DTT (200 mM) at 55℃ for 1 h to break the disulfide bond, then 5 μL of 375 mM iodoacetamide was added and incubated for 30 min to prevent the potential re-formation of the disulfide bond. The proteins were precipitated with ice-cold acetone, dissolved in 100 μL TEAB, digested with sequence-grade modified trypsin (Promega, Madison, WI), and the digested peptide mixture was labeled using chemicals from the iTRAQ reagent kit. The labeled samples were combined, desalted on a C18 SPE column (Sep-Pak C18, Waters, Milford, MA), and dried in a vacuum.

**High pH reverse-phase separation**

The peptide mixture was dissolved in buffer A (10 mM ammonium formate in water, pH 10.0, adjusted with ammonium hydroxide), and then fractionated by linear gradient high pH separation using an Aquity UPLC system (Waters Corp., Milford, MA). The column flow rate was maintained at 250 μL/min and column temperature at 45℃. Twelve fractions were collected, each of which was dried in a vacuum concentrator for the next step.

**Low pH nano-HPLC-MS/MS analysis**

The fractions were re-suspended with 40 μL solvent C (water with 0.1% formic acid D: ACN with 0.1% formic acid), separated by nanoLC, and analyzed by on-line electrospray tandem mass spectrometry. The samples were analyzed using an EASY-nLC 1000 system (Thermo Fisher Scientific, Waltham, MA) connected to an Orbitrap Fusion Mass Spectrometer (Thermo Fisher Scientific, San Jose, CA) equipped with an online nano-electrospray ion source. The samples (4 μL) were loaded onto the trap column, with a flow rate of 10 μL/min for 3 min and subsequently separated on the analytical column with a linear gradient from 5% D to 30% D in 110 min.

**Quantitative data analysis**

All proteomics sequencing and related bioinformatics analysis were performed at Biotree Biotech Co., Ltd (Shanghai, China). Briefly, the percolator algorithm was used to control the peptide level false-discovery rates <1%. Only unique peptides were used for protein quantification. Proteins contained at least two unique peptides, and the method of normalization to the median was used to correct experimental bias; the minimum number of proteins was set to 1000.

**Statistical analysis**

Data were analyzed using GraphPad Prism 5, and are presented as the mean ± SD. Multiple comparisons were analyzed by ANOVA with *post-hoc* analysis by the Newman-Keuls test. Two-tailed t-tests were used to determine the significance of differences between two groups. P <0.05 was considered statistically significant.

**References**

1. Shao, L. et al. Knockout of beta-2 microglobulin 1enhances cardiac repair by modulating exosome imprinting and inhibiting stem cell-induced immune rejection. *Cell Mol Life Sci*. **77**, 937-952 (2019).

**
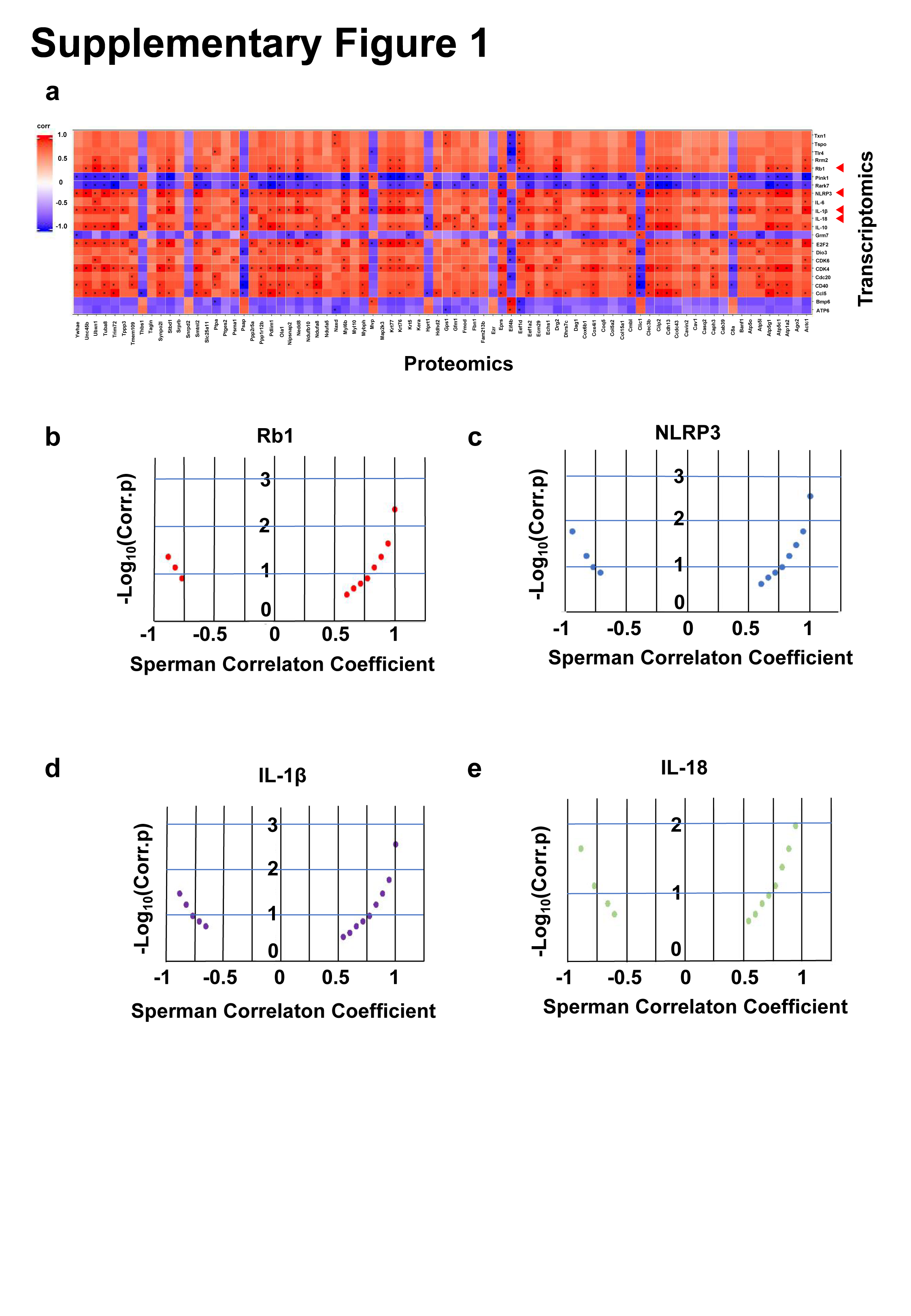
**

**Figure Legends**

**Figure. S1. The correlation between mRNA and protein-sequencing in ischemic and control muscle.**

**a** Protein-mRNA correlations analyzed by Spearman’s correlation coefficients using normalized scores (z-scores). **b-e** Spearman’s correlation analysis was performed to confirm that the increased mRNA expression of Rb1, NLRP3, IL-18, and IL-1β is correlated with the increased protein expression in ischemia compared to controls.


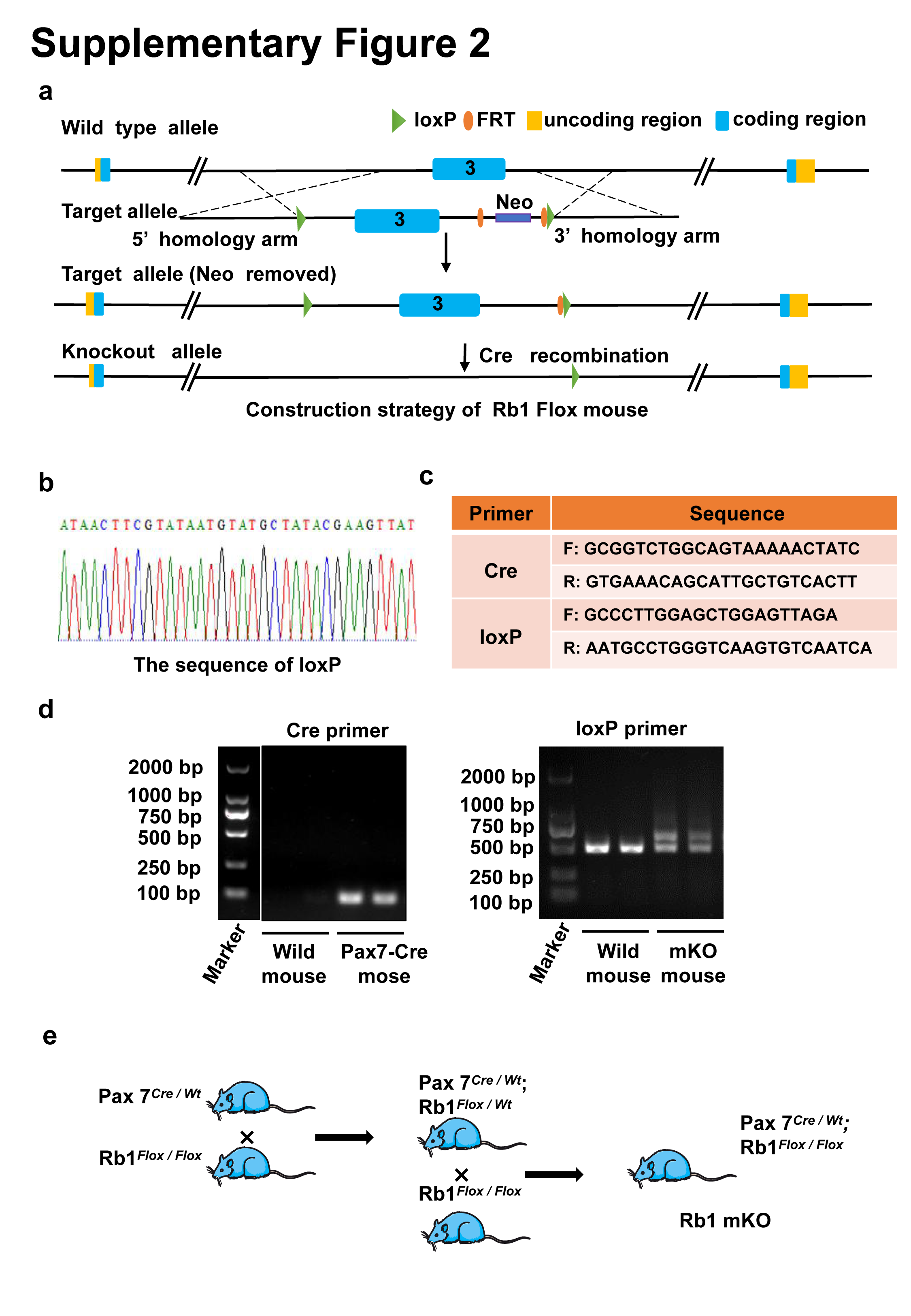


**Figure. S2. Generation of muscle specific Rb1 knockout mice.**

**a** Construction strategy of conditional Rb1 knockout mice. **b** Genetic identification of Flox mouse by DNA sequencing. **c** Mouse genotyping primers. **d** Agarose electrophoresis of PCR products of genotype identification. **e** Schematic illustration of the breeding strategy to generate muscle-specific knockout Rb1 (Rb1-mKO) mice.

**
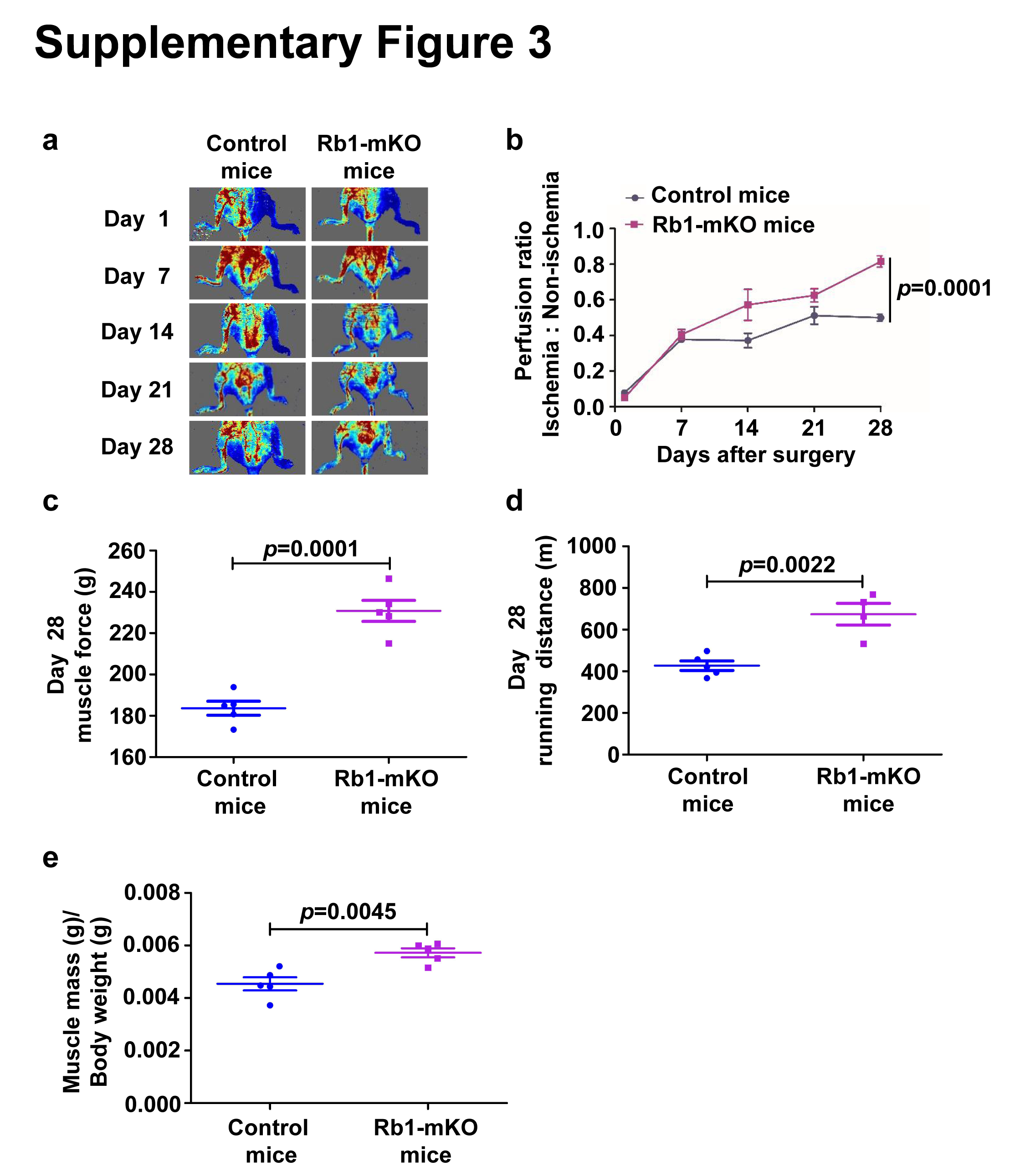
**

**Figure. S3. Knockout of the Rb1 gene promotes ischemic muscle repair.**

**a** Laser Doppler perfusion imaging of limbs from Rb1-mKO and control mice at different time points after ischemic injury. **b** Blood flow recovery after ischemic injury. **c** The muscle strength of Rb1-mKO and control mice at the day 28 after ischemic injury. **d** Running distances of Rb1-mKO and control mice at day 28 after ischemic injury. **e** Ratio of the muscle weight to the body weight 28 days after ischemic injury. Data are presented as the mean ± SD; n = 5.


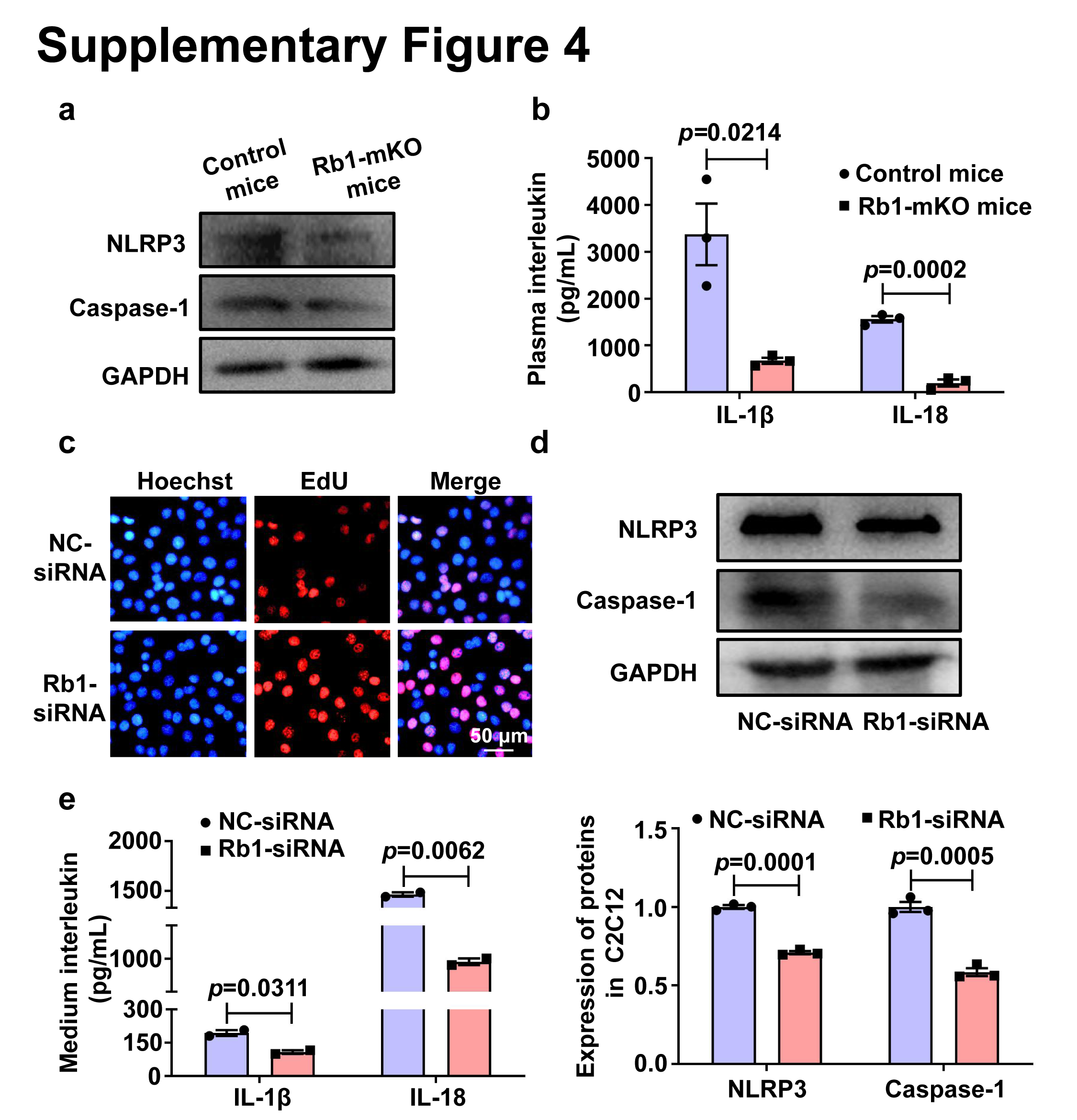


**Figure. S4. Inflammasome is inactivated by Rb1 deletion.**

**a** Western blots analysis of the expression of NLRP3 and Caspase-1 in muscles of Rb1-mKO mice. **b** ELISA analysis of the expression of IL-1β and IL-18 in plasma of Rb1-mKO mice. **c** Proliferation of C2C12 cells was determined by EdU incorporation after treatment with NC-siRNA or Rb1-siRNA. Blue, nuclear staining (Hoechst); red, EdU staining (scale bar, 50 μm). **d** Western blots showing the decreased expression of NLRP3 and Caspase-1 protein in C2C12 cells treated with Rb1-siRNA. **e** The levels of IL-1β and IL-18 from the Rb1-siRNA and NC-siRNA treated C2C12 cells were determined by ELISA. Data are presented as the mean ± SD; n = 3.


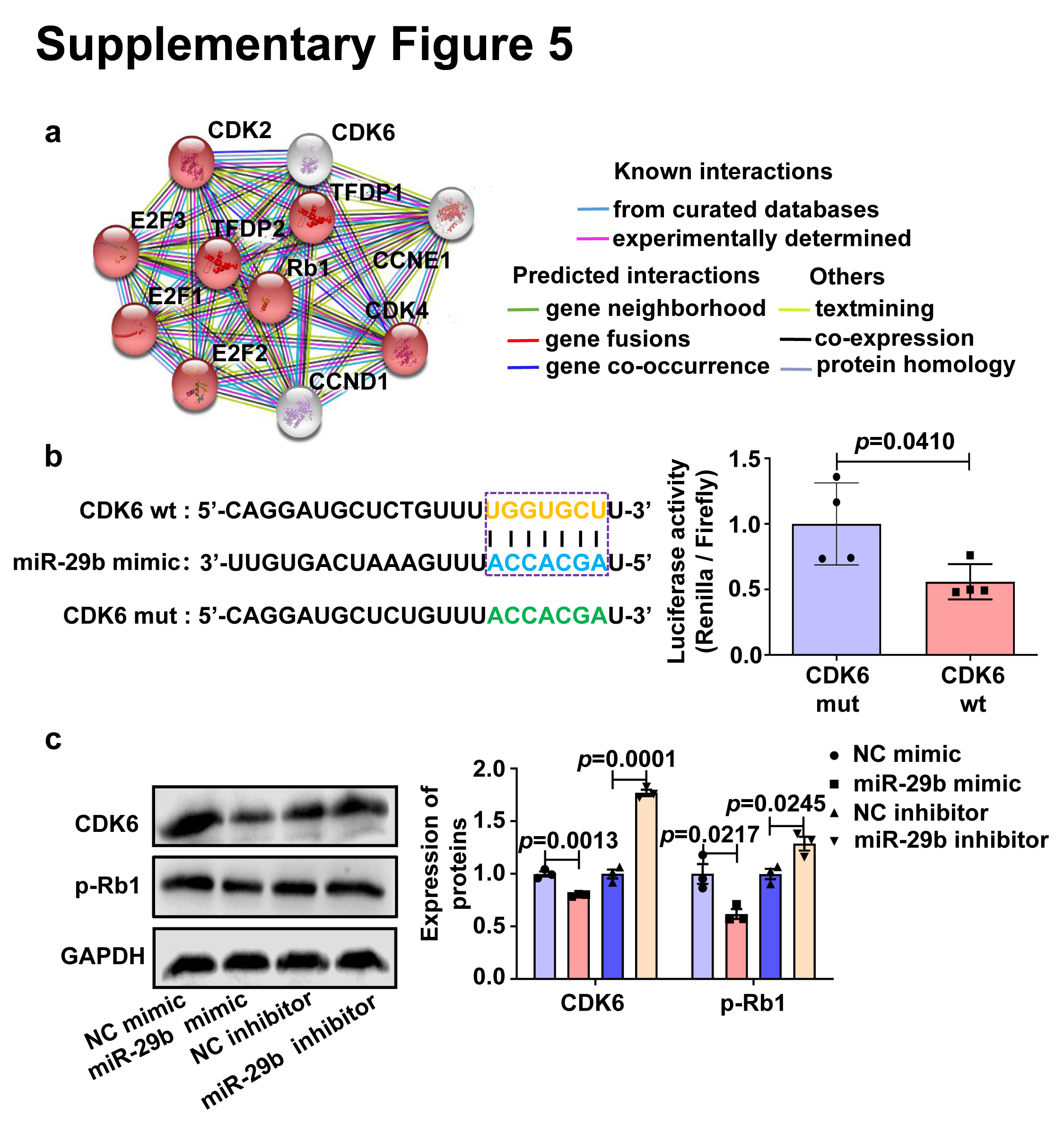


**Figure. S5. Rb1 is regulated by miR-29b and CDK6.**

**a** The interaction network of Rb1 protein was analyzed by the STRING database. **b** Targetscan analysis showing that the 3 'UTR of CDK6 mRNA and miR-29b has multiple binding sites. Dual luciferase results confirmed that CDK6 is a target of miR-29b. **c** Western blots analysis of the expression of CDK6 and p-Rb1 in C2C12 cells treated with an miR-29b mimic, or miR-29b inhibitor.


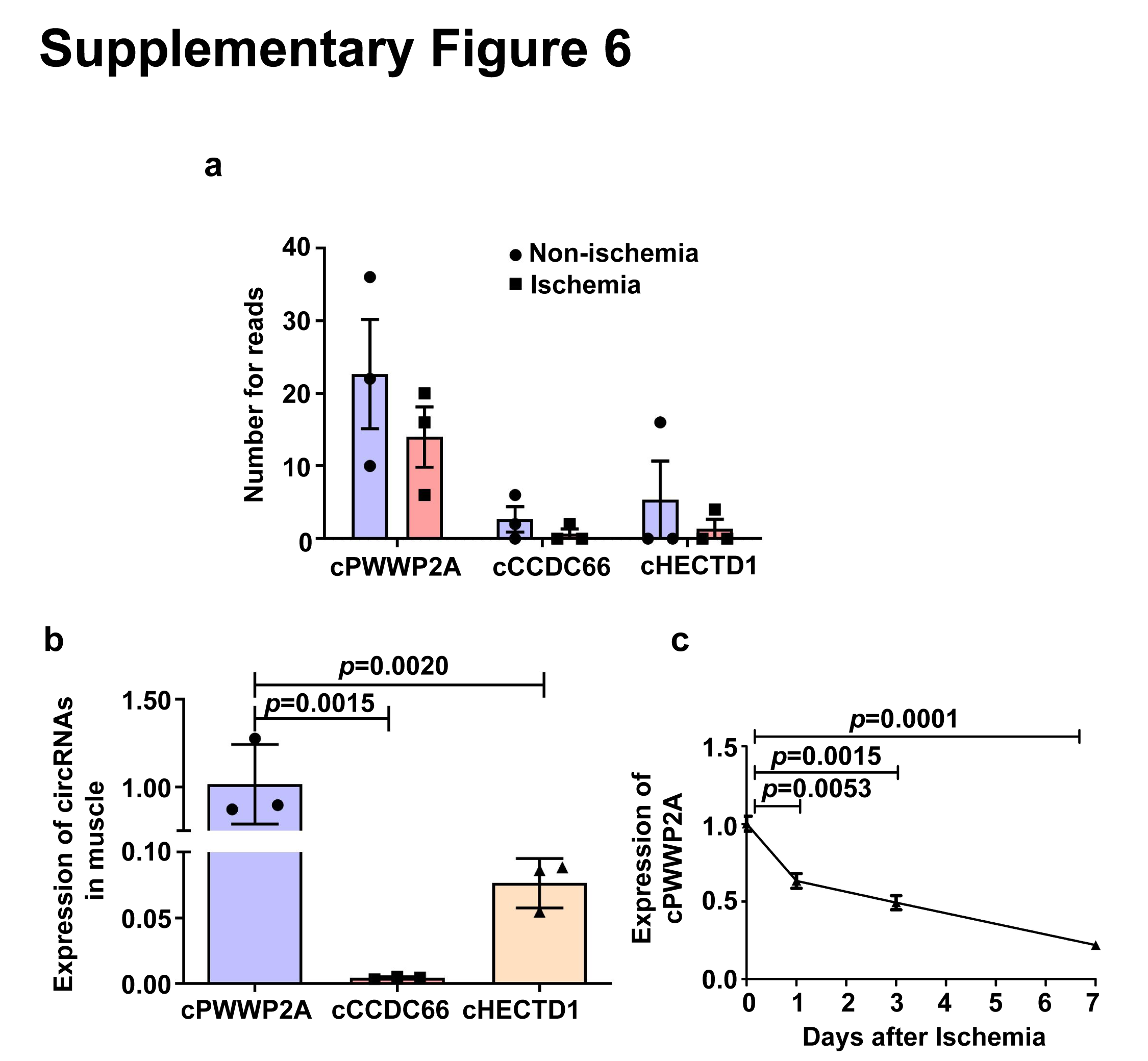


**Figure. S6. Expression of circRNAs in ischemic muscle.**

**a** Abundance of several circRNAs in muscle. **b** The expression of cPWWP2A was much higher compared to other circRNAs as determined by qRT-PCR analysis. **c** qRT-PCR analysis of cPWWP2A expression in the muscle at different time points after ischemic injury.


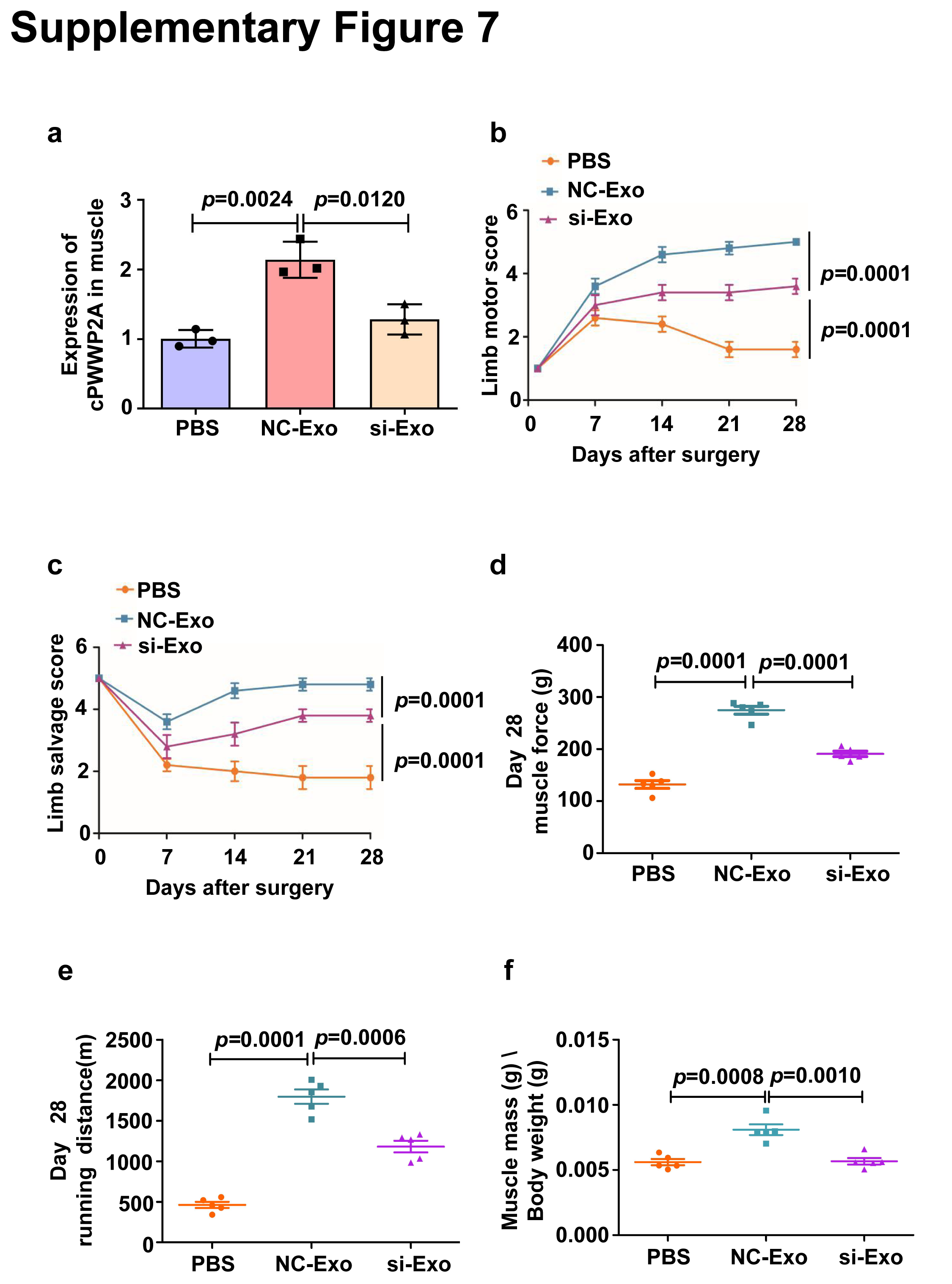


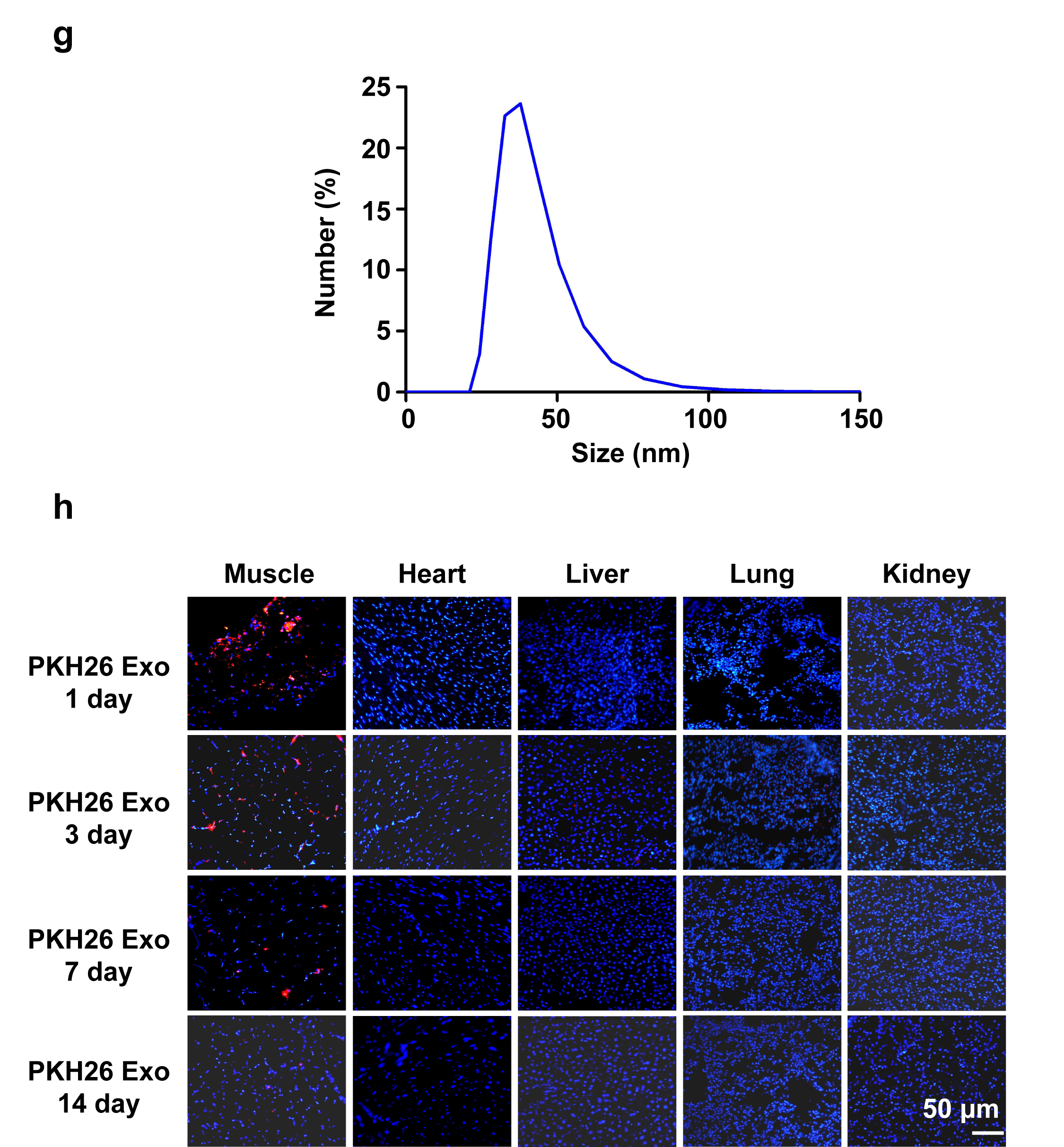


**Figure. S7. Ischemic muscle functional recovery is blunted by cPWWP2A silencing in exosomes.**

**a** Expression of cPWWP2A in muscles treated with NC-Exo, or si-cPWWP2A (si-Exo). **b** Motor function at different time points after treatment. **c** Muscle recovery in mice treated with PBS, NC-Exo, or si-Exo at different time points. **d** Muscle strength at 28 days after treatment. **e** Running distance of mice after different treatments. **f** Ratio of muscle weight to the body weight at 28 days after PBS, NC-Exo, or si-Exo treatment. **g** Nanosight analysis of the diameter of exosomes. **h** After PKH26-labeled exosomes were injected into the muscles, the biodistribution of exosomes were investigated by analyzing PKH26 signals in frozen sections of muscle, heart, liver, lung and kidney using a fluorescent microscope. Data are presented as the mean ± SD; n = 5.

**
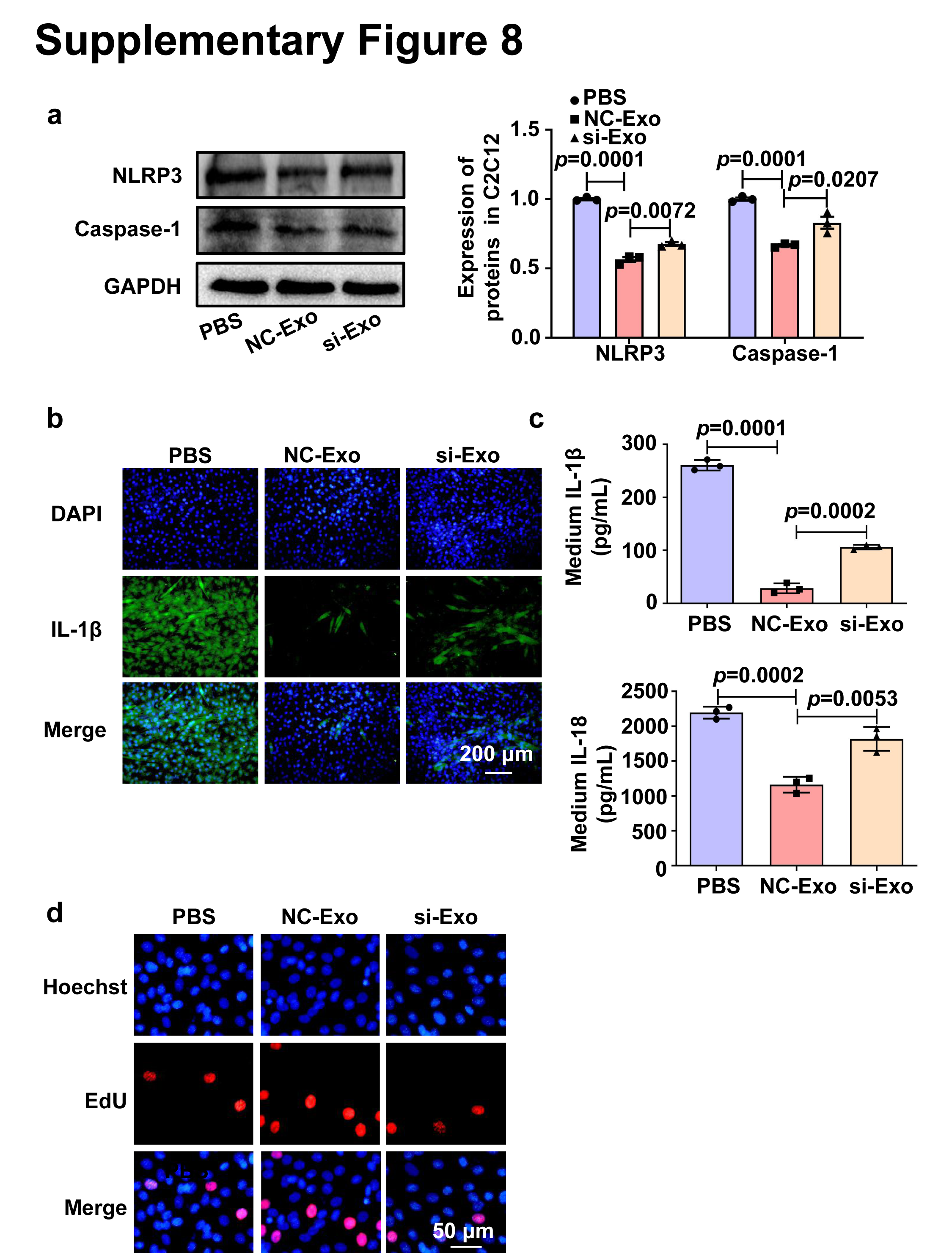
**

**Figure. S8. cPWWP2A inhibits inflammasome activation in C2C12 cells.**

**a** Western blot analysis of the expression of NLRP3 and Caspase-1 proteins in C2C12 cells treated with PBS, NC-Exo, or si-Exo. **b, c** Immunofluorescence staining and ELISA analysis of IL-1β and IL-18 levels in C2C12 cells pre-treated with PBS, NC-Exo, or si-Exo. **d** Proliferation of C2C12 cells was determined by EdU incorporation after the cells were treated with PBS, NC-Exo, or si-Exo. Blue, nuclear staining (Hoechst); red, EdU staining (scale bar, 50 μm). Data are presented as the mean ± SD; n = 3.

**
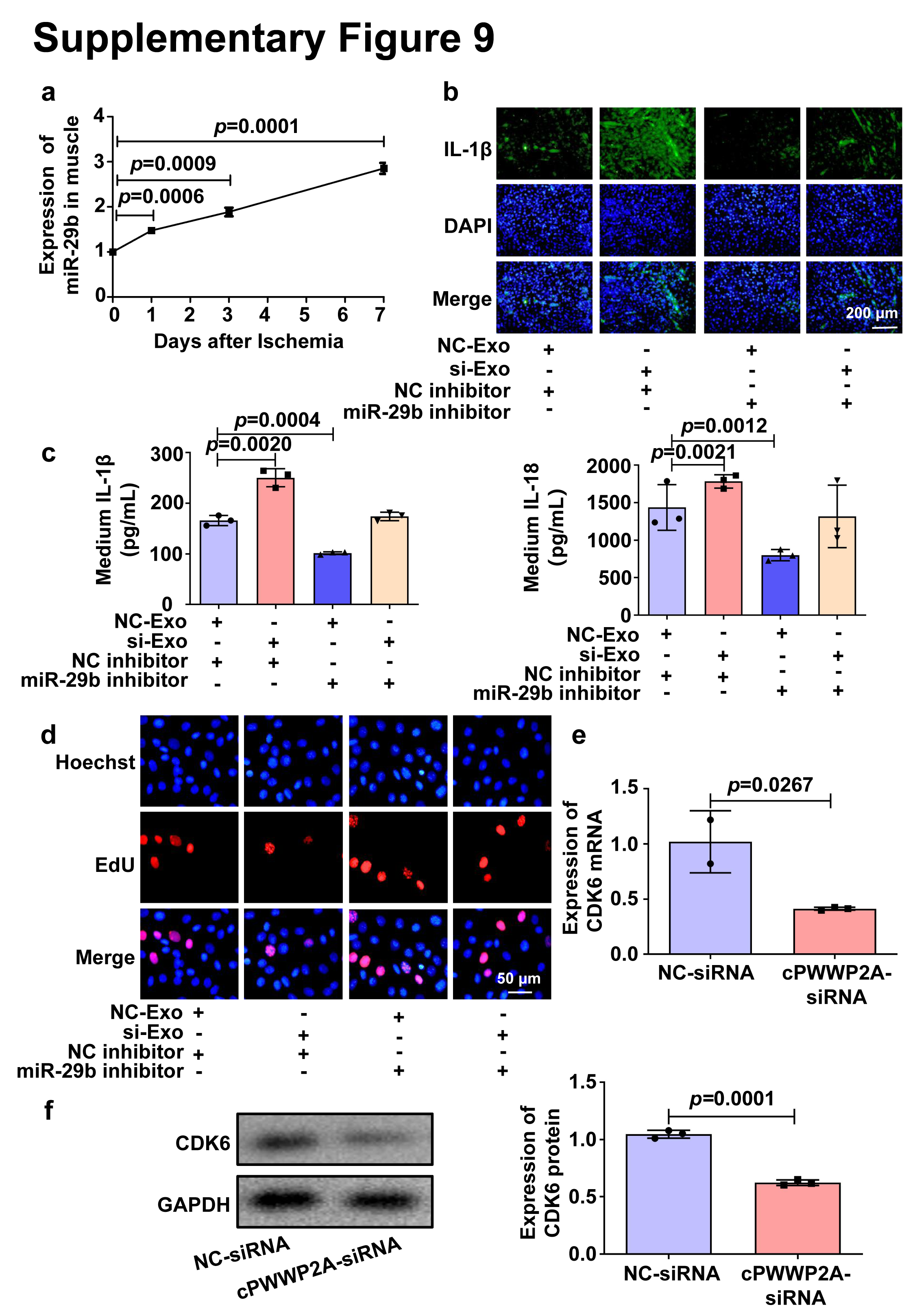

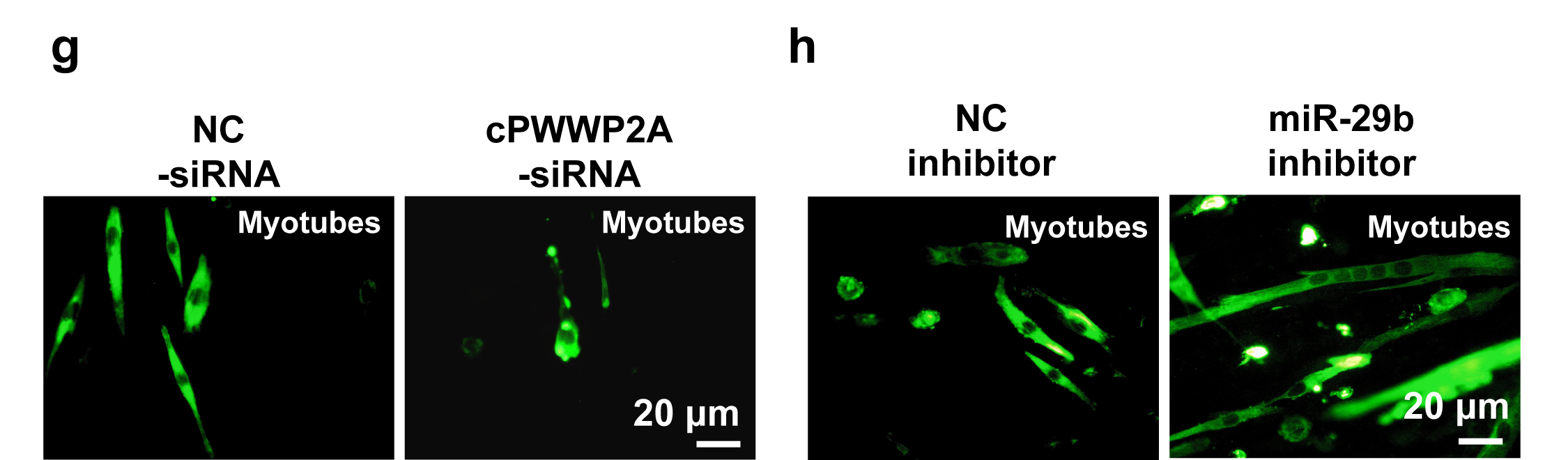
**

**Figure. S9. cPWWP2A/miR-29b regulates the NLRP3 signaling pathway.**

**a** qRT-PCR analysis showing increased levels of miR-29b in the muscle at different time points after ischemic injury. **b, c** Immunofluorescence staining and ELISA analysis showing that inhibition of miR-29b in C2C12 cells reduces the levels of IL-1β and IL-18, and the effects are reversed by cPWWP2A-silencing. **d** The miR-29b inhibitor enhances C2C12 cell proliferation, and this effect is reversed by cPWWP2A silencing. **e** qRT-PCR analysis of mRNA expression of CDK6 in C2C12 cells treated with cPWWP2A siRNA. **f** Western blots analysis of the expression of CDK6 protein in C2C12 cells treated with cPWWP2A siRNA. **g** Immunofluorescent staining of myotubes. C2C12 cells were transfected with either cPWWP2A siRNA or NC-siRNA and cultured in differentiation medium for 4 days. The myotubes were immunostained with MF-20 antibody. **h** C2C12 cells were transfected with either miR-29b inhibitor or NC-inhbitor, and cultured in differentiation medium for 4 days. The myotubes were immunostained with MF-20 antibody. Data are presented as the mean ± SD; n = 3.


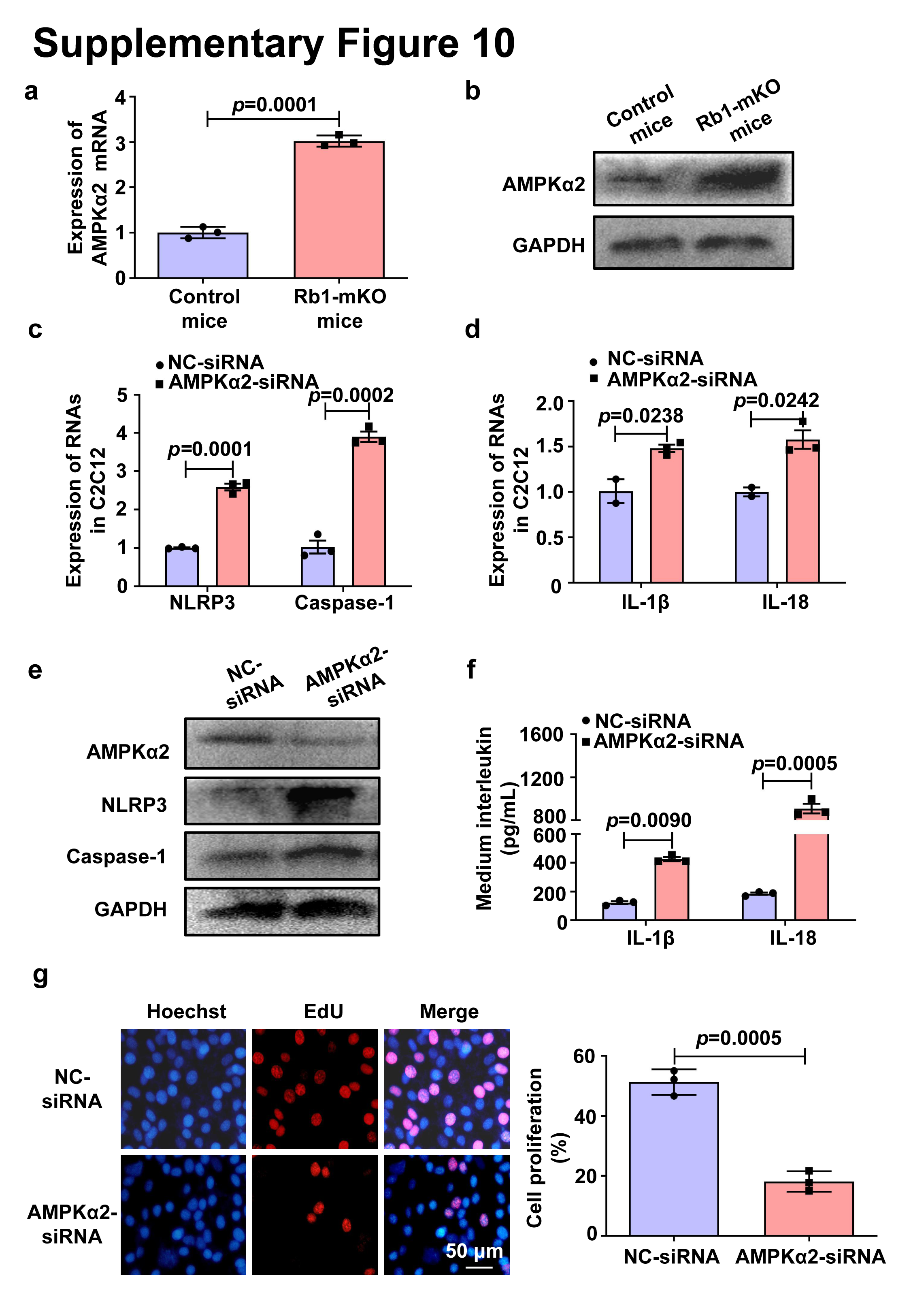


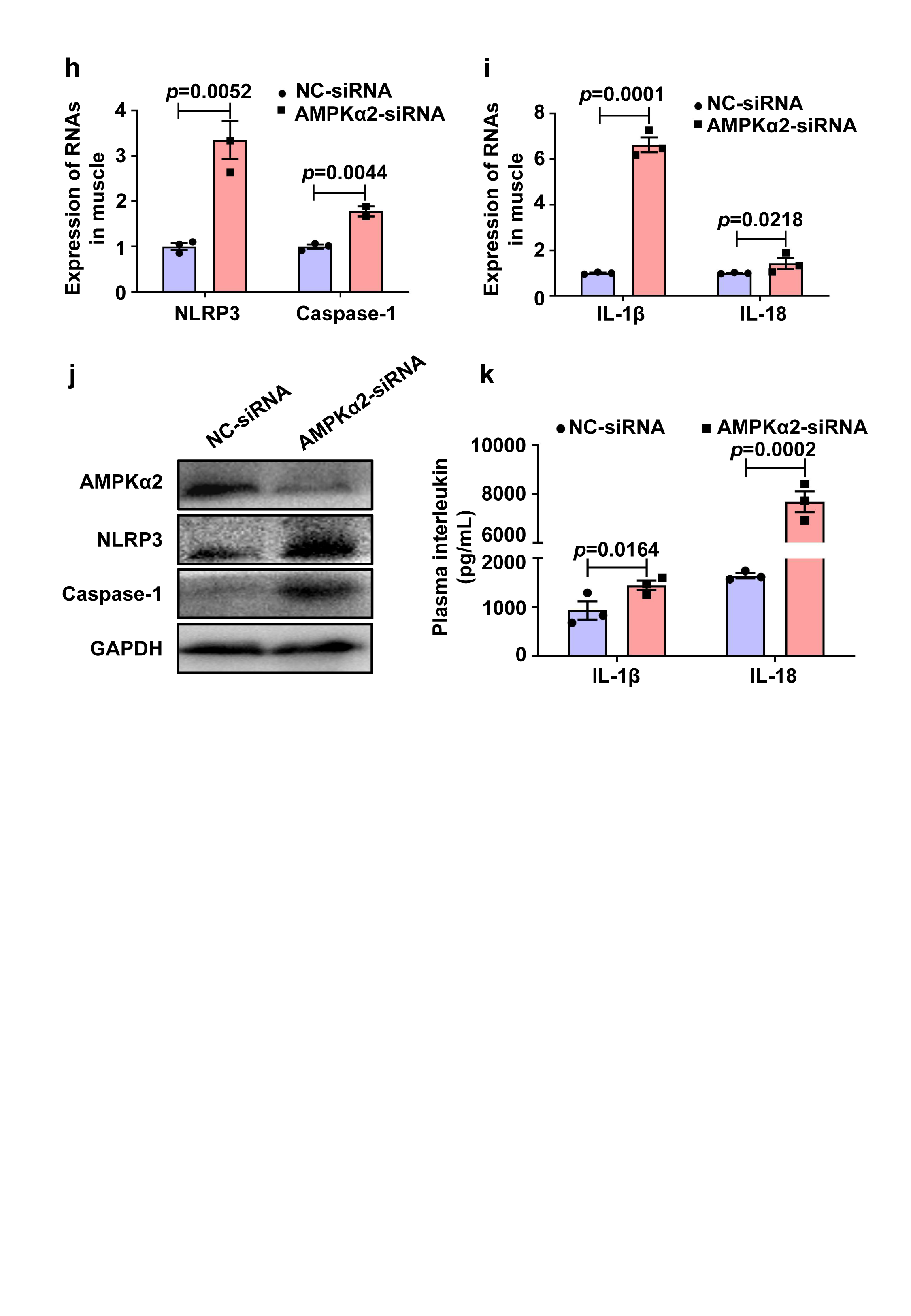


**Figure. S10. Rb1 inhibits the activation of NLRP3 inflammasome by regulating the expression of AMPKα2.**

**a, b** qRT-PCR and Western blot analysis of the expression of AMPKα2 in muscles from Rb1-mKO mice. **c, d** qRT-PCR analysis of the mRNA expression of NLRP3, Caspase-1, IL -1β and IL-18 in C2C12 cells treated by AMPKα2 siRNA. **e, f** Western blot and ELISA analysis of the expression of NLRP3, Caspase-1, IL -1β and IL-18 in C2C12 cells treated by AMPKα2 siRNA. **g** The effect of AMPKα2 siRNA on C2C12 cell proliferation was assessed by EdU staining. **h, i** qRT-PCR analysis of the mRNA expression of NLRP3, Caspase-1, IL -1β and IL-18 in muscle treated by AMPKα2 siRNA. **j** Western blot analysis of AMPKα2, NLRP3 and Caspase-1 in muscles treated with AMPKα2 siRNA or NC-siRNA. **k.** ELISA analysis of plasma levels of IL -1β and IL-18 in mice treated with AMPKα2 siRNA or NC-siRNA. Data are presented as the mean ± SD; n = 3.
